# *Caenorhabditis elegans* is a useful model for anthelmintic discovery

**DOI:** 10.1038/ncomms8485

**Published:** 2015-06-25

**Authors:** Andrew R. Burns, Genna M. Luciani, Gabriel Musso, Rachel Bagg, May Yeo, Yuqian Zhang, Luckshika Rajendran, John Glavin, Robert Hunter, Elizabeth Redman, Susan Stasiuk, Michael Schertzberg, G. Angus McQuibban, Conor R. Caffrey, Sean R. Cutler, Mike Tyers, Guri Giaever, Corey Nislow, Andy G. Fraser, Calum A. MacRae, John Gilleard, Peter J. Roy

**Affiliations:** 1 The Donnelly Centre for Cellular and Biomolecular Research, University of Toronto, Toronto, Ontario, Canada M5S 3E1; 2 Department of Molecular Genetics, University of Toronto, Toronto, Ontario, Canada M5S 1A8; 3 Cardiovascular Division, Brigham and Women's Hospital, Harvard Medical School, and Harvard Stem Cell Institute, Boston, Massachusetts 02115, USA; 4 Department of Medicine, Harvard Medical School, Boston, Massachusetts 02115, USA; 5 Department of Comparative Biology and Experimental Medicine, Faculty of Veterinary Medicine, University of Calgary, Calgary, Alberta, Canada T2N 4Z6; 6 Department of Biochemistry, University of Toronto, 1 King's College Circle, Toronto, Ontario, Canada M5S 1A8; 7 Center for Discovery and Innovation in Parasitic Diseases and Department of Pathology, University of California, San Francisco, California 94158, USA; 8 Center for Plant Cell Biology, Department of Botany and Plant Sciences, University of California, Riverside, California 92521, USA; 9 Institute for Research in Immunology and Cancer, University of Montreal, Montreal, Quebec, Canada H3T 1J4; 10 Department of Pharmaceutical Sciences, University of British Columbia, Vancouver, British Columbia, Canada V6T 1Z3; 11 Department of Pharmacology and Toxicology, University of Toronto, Toronto, Ontario, Canada M5S 1A8

## Abstract

Parasitic nematodes infect one quarter of the world's population and impact all humans through widespread infection of crops and livestock. Resistance to current anthelmintics has prompted the search for new drugs. Traditional screens that rely on parasitic worms are costly and labour intensive and target-based approaches have failed to yield novel anthelmintics. Here, we present our screen of 67,012 compounds to identify those that kill the non-parasitic nematode *Caenorhabditis elegans*. We then rescreen our hits in two parasitic nematode species and two vertebrate models (HEK293 cells and zebrafish), and identify 30 structurally distinct anthelmintic lead molecules. Genetic screens of 19 million *C. elegans* mutants reveal those nematicides for which the generation of resistance is and is not likely. We identify the target of one lead with nematode specificity and nanomolar potency as complex II of the electron transport chain. This work establishes *C. elegans* as an effective and cost-efficient model system for anthelmintic discovery.

## Abstract (web summary)

Screening for new anthelmintic compounds that are active against parasitic nematodes is costly and labour intensive. Here, the authors use the non-parasitic nematode *Caenorhabditis elegans* to identify 30 anthelmintic lead compounds in an effective and cost-efficient manner.

## Graphical abstract

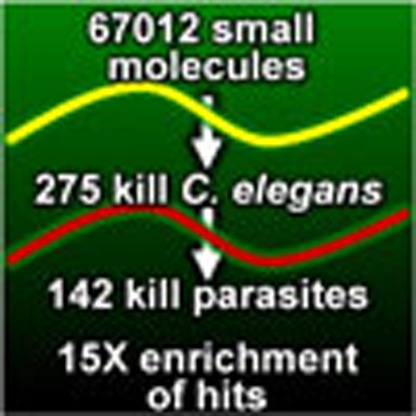

Parasitic nematodes are estimated to infect about one quarter of all humans and have a dramatic negative impact on human health and productivity in developing nations^1^,^2^. Nematode infections of agriculturally important plants and animals also result in huge economic losses worldwide^3^,^4^. Despite this, only a handful of anthelmintic families are currently available. These include the benzimidazoles, macrocyclic lactones (for example, ivermectin), imidazothiazoles (for example, levamisole) and cyclic octadepsipeptides (for example, emodepside), most of which were introduced decades ago. Nematode resistance has been reported for each class of compound, with some natural isolates showing multidrug resistance^3^,^5^. Anthelmintic resistance is a global issue; although some regions, such as New Zealand, have a particularly high prevalence of resistant parasites^5^. Amino-acetonitrile derivatives (AADs) such as monepantel have recently been introduced to the market; however, resistance to this class of compounds has already been reported^6^,^7^,^8^. While combinatorial strategies may prolong an anthelmitic's utility, growing resistance poses significant challenges for the management of parasitic infections.

One reason for the limited number of available anthelmintics may be related to the difficulty in identifying lead compounds at high throughput. The complex life cycle of parasitic nematodes, which rely on a host for propagation, make it challenging to examine a small molecule's impact on these animals with the throughput required to identify large numbers of candidate molecules for further development. The free-living nematode *Caenorhabditis elegans* may offer a convenient alternative model system to search for new compounds that specifically kill nematodes. *C. elegans*, which is ∼1 mm in length as an adult, can be cultured in high-throughput format for multiple generations, allowing the identification of molecules that perturb the worm at any point during its life cycle^9^,^10^,^11^,^12^.

The majority of marketed anthelmintics are active against *C. elegans*^6^,^13^, and the use of this model system has been instrumental in improving the understanding of the mechanism of action of several anthelmintic compounds, including levamisole, benzimidazole and the amino-acetonitrile derivatives^6^,^13^,^14^. Notably, the targets of each of these compounds have been elucidated through forward genetic screens for *C. elegans* mutants that resist their effects. In these screens, *C. elegans* parents are randomly mutagenized and their progeny are subsequently screened for individuals that can resist the effects of a given bioactive molecule. ‘Drug'-resistant strains are analysed genetically to identify the resistance-conferring mutant gene. The most frequent resistance-conferring mutant gene within the collection of resistant strains has been shown to encode either the target or the targeted pathway/complex of these bioactive molecules^6^,^9^,^14^.

Clearly, *C. elegans* is a useful model system to study anthelmintics and offers throughput that is not possible with parasitic species. It therefore follows that it might also be a powerful system with which to screen for anthelmintic lead compounds, as has been suggested over 30 years ago^15^. However, there have only been anecdotal references to the use of *C. elegans* in the anthelmintic screening programs of the pharmaceutical industry^16^,^17^,^18^,^19^,^20^, the details of which have not been publically described. Thus, whether bioactivity in *C. elegans* is generally predictive of bioactivity in parasitic nematode species remains unknown.

Here, we describe our screen for anthelmintic lead compounds using whole *C. elegans* nematodes as the primary model system. We screened 67,012 distinct small molecules for their ability to kill *C. elegans* and re-screened our hits in two widely studied parasitic nematode models: *Cooperia onchophora*, a parasite of cattle, and *Haemonchus contortus*, a parasite of sheep^5^,^21^. We counter-screened the nematicidal molecules in two vertebrate models of development (HEK293 cells and zebrafish) and identified a set of molecules that kills nematodes but may be inactive in vertebrates. In an effort to identify the protein targets for 39 nematicides, we screened more than 19 million mutant *C. elegans* for resistance. We identified the target of one family of these lethal molecules that is closely related to nematicides that have recently been introduced to the market^22^,^23^, demonstrating the value of *C. elegans* as a model system for the discovery of useful nematicidal molecules.

## Results

### Molecules that kill *C. elegans* are likely to kill parasites

**Figure 1:**
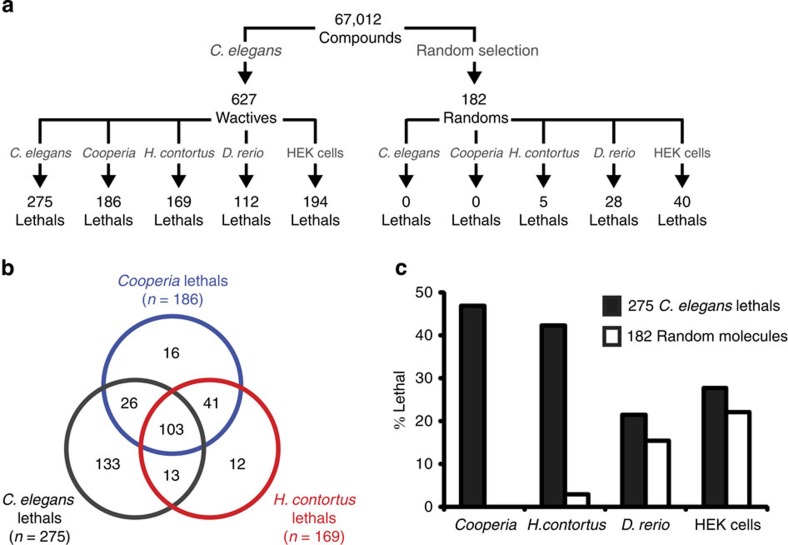
Molecules that kill *C. elegans* are enriched for those that are lethal to parasitic nematodes. (**a**) Flow chart outlining the multi-organism small-molecule screening pipeline. (**b**) Venn diagram showing the overlap of wactive library molecules that kill *C. elegans*, *Cooperia oncophora* and *H. contortus*. (**c**) Chart showing the enrichment of molecules that kill *Cooperia*, *H. contortus*, zebrafish, and HEK cells in the set of 275 *C. elegans*-lethals, relative to a randomly selected set of 182 compounds.

To identify nematicidal compounds, we screened 67,012 commercially available small drug-like molecules for those that induce obvious phenotypes in *C. elegans* at a concentration of 60 μM or less (see Fig. 1a and Supplementary Data 1). From our preliminary screens, we identified 627 bioactive molecules that we call ‘worm actives' or ‘wactives'. Rescreening revealed 275 wactives that kill *C. elegans* at a concentration of 60 μM or less (see Supplementary Data 1). By contrast, none of the 182 molecules chosen at random from the set of 67,012 compounds killed *C. elegans* (see Supplementary Data 1).

We next screened the wactive library against the nematode parasites *C. onchophora* and *H. contortus* (Fig. 1a). We chose these two species, both of which are from the same phylogenetic clade as *C. elegans* (clade V), because we could screen them using similar methods that we used to screen *C. elegans* and because many important parasites of humans and domestic livestock are from clade V. We collected nematode eggs from infected animals and tested whether the wactives could kill the eggs or hatched animals (see Methods). Of the 275 wactives that killed *C. elegans*, 129 and 116 killed at least 90% of the *C. onchophora* and *H. contortus* animals, respectively, and 103 killed all three nematode species (Fig. 1b). Of the 182 randomly chosen molecules, none killed *C. onchophora* and five killed *H. contortus*. Hence, molecules that kill *C. elegans* are more than 15 times more likely to kill these parasitic nematodes compared with randomly chosen molecules (Fig. 1c).

We counter-screened the wactive and random control libraries for activity against two vertebrate models: *Danio rerio* (zebrafish) and HEK293 cells (Fig. 1a). Fifty-nine of the 275 wactives that kill *C. elegans* and 28 of the random molecules either kill or cause substantial morbidity in zebrafish (see Methods), representing an enrichment of <1.4-fold (Fig. 1c). Similarly, 76 of the 275 wactives that kill *C. elegans* and 40 of the random molecules caused substantial growth defects in HEK293 cells (see Methods), representing an enrichment of <1.3-fold (Fig. 1c). These results suggest that *C. elegans* is a useful model system with which to identify molecules that are lethal in parasitic nematodes, without generally being cytotoxic in vertebrates.

On analysis of chemical properties, we found that nematicidal compounds had a higher average computed octanol/water partition coefficient (logP; 3.9 versus 3.2, *P*<10^−13^; Student's *t*-test) and lower average molecular weight (273 versus 328, *P*<10^−20^; Student's *t*-test) when compared against the complete set of 67,012 compounds (Supplementary Fig. 1). This suggests that molecules that are smaller and with greater lipid-solubility might be more effective nematicides.

### Structure analyses reveal 30 classes of anthelmintic leads

**Figure 2:**
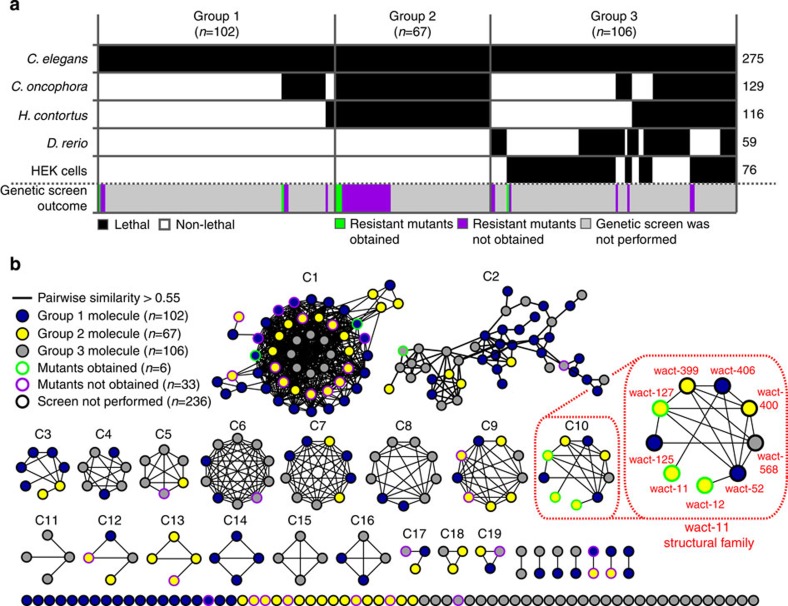
Nematode selectivity and structural profiling of the 275 *C. elegans*-lethal molecules. (**a**) Heat map indicating the lethality (or lack thereof) induced by each of the 275 *C. elegans*-lethals in two species of parasitic nematode, as well as zebrafish embryos and human embryonic kidney (HEK) cells. For each species, the number of molecules that induce lethality is indicated to the right of the heat map. The molecules segregate into three groups based on their nematode selectivity and cross-species lethality. If a genetic screen for resistant mutants was performed for a given molecule, this is indicated, as well as the outcome of the screen. (**b**) Network based on the structural similarity of the 275 *C. elegans*-lethal molecules. Nodes represent molecules, and edges connect molecules with a pairwise Tanimoto/FP2 score >0.55 (see Methods). The group to which each molecule belongs is indicated by the node fill colour, whereas the genetic screen information is indicated by the node border colour. In the legend, the number of molecules is indicated in parentheses. The 19 clusters containing three or more molecules are named C1 to C19. The wact-11 structural family (cluster C10) is magnified, and the names of each molecule in the family are indicated.

To further characterize our 275 *C. elegans*-lethal molecules, we organized them into three separate groups based on their phylogenetic bioactivity profiles (Fig. 2a). Group 1 contains 102 molecules that are lethal to only one or two of the three nematode species tested, but are non-lethal to zebrafish and HEK cells. Group 2 contains 67 compounds that are lethal to all three nematode species, but are non-lethal to zebrafish and human cells. Group 3 contains the remaining 106 compounds that are lethal to fish or HEK cells, and have varied bioactivity in different nematode species. In particular, the 67 molecules in group 2 represent potentially ideal anthelmintic leads; they have activity across multiple nematode species, and appear not to affect vertebrates.

To better understand the structural relationships that exist among our *C. elegans*-lethal compounds, we constructed a structure similarity network that connects molecules if they have a pairwise Tanimoto/FP2 similarity >0.55 (Fig. 2b; see Methods). This network contains 19 isolated clusters composed of three molecules or more, leaving 72 unconnected singletons or pairs. Each cluster represents a unique structural family that could, in principle, target a single protein, although the larger C1 and C2 clusters may contain multiple subfamilies of structures. The 67 group 2 molecules are distributed across 12 clusters, two pairs and 16 singletons, representing 30 structurally unique classes of anthelmintic leads that may target 30 or more distinct protein targets.

To estimate our ability to improve upon the biological activity of the 67 group 2 molecules through medicinal chemistry, we compared the structural similarity of each of these wactives with the 67,012 molecules that we screened (see Methods; Supplementary Data 1). We reasoned that the fraction of molecules within a family of structural analogues that are bioactive may be predictive of the ability to create novel analogues that are bioactive, some of which may have improved bioactivity. We found that 38 (57%) of the 67 wactives are members of a structural analogue family (based on a pairwise Tanimoto/FP2 similarity score of 0.55 or greater) for which more than 10% are lethal to *C. elegans* (Supplementary Fig. 3). Considering only the 18 group 2 wactives that are excluded from the 19 structural clusters in Fig. 2b, we found that 10 (56%) are members of a structural analogue family for which more than 10% are lethal to *C. elegans*. These results suggest that many of the group 2 wactives have the potential for improvement through medicinal chemistry efforts.

### Modest structural changes impact phylogenetic specificity

The C3, C13 and C14 clusters in Fig. 2b are composed exclusively of molecules that selectively kill nematodes, suggesting that these families may be targeting nematode-specific proteins. The remaining 16 structural families contain at least one molecule that is lethal to either fish or human cells, suggesting that if the compounds in a cluster target a single protein, the target might be conserved in vertebrates. At the very least, these 16 clusters suggest that relatively modest structural changes can alter the species selectivity of the molecules in a family. To further explore the divergence of species selectivity within these 16 structural families, we focused on pairs of molecules that have a Tanimoto/FP2 pairwise similarity score of 80% or more, and for which one molecule in the pair specifically kills nematodes (group 2 molecules) and the other kills both nematodes and a vertebrate model (group 3 molecules). Seventeen pairs of molecules satisfy these criteria (Supplementary Fig. 2). Inspection of the structural differences of the individual molecules within each pair reveals that very small structural changes can restrict a molecule's bioactivity to nematodes. For example, wact-1, wact-433 and wact-434 in cluster 18 are identical except that an ethyl group in wact-434 replaces the halogen of wact-1 and wact-433. The halogen substitution destroys the core molecule's bioactivity in fish and restricts its activity to nematodes. Medicinal chemistry often yields structural analogues that have reduced or abolished bioactivity. However, our structure–activity analysis has revealed analogues that have not lost bioactivity but have instead become phylogenetically restricted.

### Genetic resistance to most nematicides is not easily induced

Forward genetic screens with *C. elegans* have been previously used to identify the targets of bioactive compounds^6^,^9^,^14^. Here, we chose 39 compounds from the set of 275 *C. elegans*-lethal wactives to pursue their target genetically (see Methods; Supplementary Data 1; Fig. 2). These 39 molecules span 19 distinct structural classes (Fig. 2b), and the majority of these compounds are found in group 2, which are lethal to all three nematodes tested and non-lethal to zebrafish and HEK293 cells (Fig. 2a).

To identify mutants that are resistant to specific compounds, we randomly mutagenized wild-type *C. elegans* parental (P0) worms using chemical mutagens (see Methods) and screened for animals that resist lethality in either the first (F1) or second (F2) generation. Resistant mutants that arise in F1 screens are dominant and typically encode missense mutations that confer resistance against antagonists, while resistance-conferring mutations in F2 animals are typically recessive reduction-of-function mutations that confer resistance to agonists^24^. Molecules against which F1 screens did not yield resistant mutants were screened again for F2-resistant mutants. We performed F1 screens for all 39 lethal molecules, and F2 screens for 29 of the 39 compounds (Supplementary Table 1). In total, we screened over 19 million mutant genomes and we were able to isolate resistant mutants against only six of the 39 molecules (Supplementary Table 1).

### Wact-11-resistant mutants show intra-family cross-resistance

**Figure 3:**
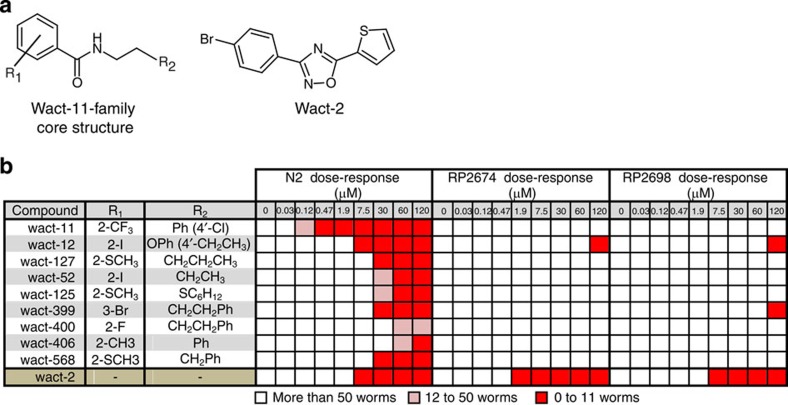
Wact-11 and wact-12 resistant mutants are cross-resistant to all nine wact-11-family members. (**a**) The wact-11-family core structure and the structure of an unrelated molecule, wact-2, which was used as a negative control throughout this work. (**b**) Heat maps of the wact-11-family dose-response experiments with wild-type worms (N2 strain), as well as two mutant strains, RP2674 and RP2698, isolated as being resistant to wact-12 and wact-11, respectively. The dose-response experiments were carried out using a 96-well plate liquid-based assay (see Methods). White indicates that there were more than 50 worms in three out of four replicate wells. Pink indicates that there were between 12 and 50 worms in three out of four replicate wells. Red indicates that there were between 0 and 11 worms in three out of four replicate wells. In the case of ties, the higher number prevailed (for example, at a given concentration, if two wells had 55 worms, and the other two wells had 20 worms, the chemical would be scored as having more than 50 worms). The R_1_ and R_2_ groups are indicated for each wact-11-family member. Wact-2 is used here as a negative control.

Wact-11, wact-12 and wact-127 are three of the molecules against which we could generate resistant mutants (Supplementary Table 1). These three molecules are part of the C10 cluster (Fig. 2b), which we refer to as the ‘wact-11 family', and share an ethyl benzamide moiety (Fig. 3). In total, we isolated 37 mutants that resist the wact-11 family members at a rate of one mutant per 100,000 genomes screened (Supplementary Table 1). Using a representative set of 21 mutants, we performed a detailed dose-response analysis of each mutant against wact-11 and the structurally unrelated nematicide wact-2 in liquid media (Supplementary Fig. 4). All of the tested mutants show at least some resistance to wact-11, but not to wact-2, indicating that they are specifically resistant to wact-11.

Given their structural similarity, we hypothesized that each compound within the wact-11 family may share the same mechanism of action. If true, then mutants that were isolated based on their resistance to one wact-11 family member will resist the lethality that is induced by other family members. To test this, we performed a dose-response analyses of two wact-11-family resistant mutants (isolated based on their respective resistance to wact-11 and wact-12) against all nine wact-11-family members. Both mutants were resistant to all nine wact-11-family members (Fig. 3), supporting the idea that all nine members of the wact-11 family act by the same mechanism.

### The wact-11 family inhibits worm mitochondrial complex II

To identify candidate targets of the wact-11-family, we sequenced the genomes of 33 resistant mutants to identify mutated genes common to multiple strains (Supplementary Data 2 and 3). Ten strains had missense mutations in *sdhb-1*, a different set of 16 strains had a missense mutation in *sdhc-1* (otherwise known as *mev-1*), six other strains had missense mutations in *sdhd-1* and one remaining strain had no commonly mutated gene (Table 1). By contrast, no other gene is represented by distinct mutant alleles in more than four of the 33 strains, and none of the *sdh* genes are mutated in 36 strains that resist the effects of two other unrelated molecules (A.R.B., Houtan Moshiri and P.J.R., unpublished results). Consistent with the isolation of the majority of these mutants in F1 screens, none of the *sdh* mutations are nonsense, frame-shifts or deletions that would be indicative of loss-of-function. Instead, the missense mutations change four unique residues in SDHB-1, seven unique residues in SDHC-1 and three unique residues in SDHD-1 (Table 1).

SDHB-1, SDHC-1 and SDHD-1, along with SDHA-1, are the four protein subunits of *C. elegans* mitochondrial complex II^25^ (otherwise known as succinate dehydrogenase or *sdh*), which couples the citric acid cycle to the electron transport chain and is highly conserved among eukaryotes^26^. Complex II couples the oxidation of succinate to fumarate, with the reduction of ubiquinone to ubiquinol^26^. Eukaryotic complex II has at least one ubiquinone-binding site, referred to as the Qp site or Q-site, that exists at the intersection of the SDHB, SDHC and SDHD subunits. In contrast, the succinate-binding site is found exclusively in the SDHA subunit.

**Figure 4:**
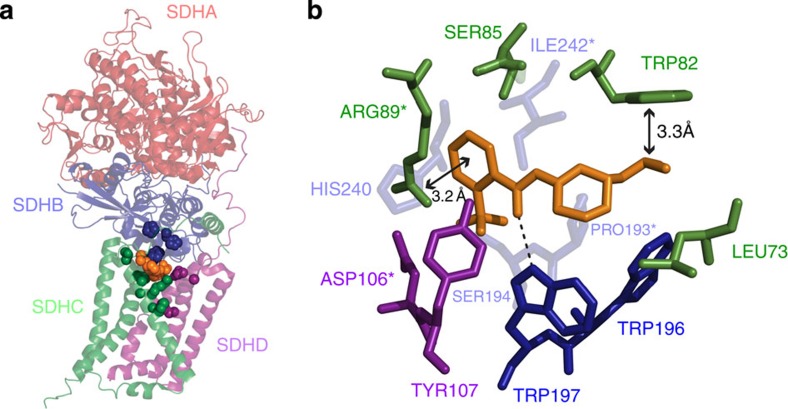
Complex II residues that are mutated in the wact-11 family resistant mutants cluster near the ubiquinone-binding site (Q-site). (**a**) Rendering of the crystal structure of *Ascaris suum* Complex II bound to the Q-site inhibitor flutolanil (PDB: 3VRB). The side chains of the 14 orthologous residues that are mutated in the wact-11-family resistant mutants are shown as opaque spheres. The atoms of the bound flutolanil molecule are shown as orange-coloured opaque spheres. (**b**) Close-up view of flutolanil bound at the Q-site of Complex II from *Ascaris suum*. The 12 residues shown are no more than 4 Å away from flutolanil, and make up the flutolanil binding pocket. Intermolecular distances are indicated with bidirectional arrows. The dashed line represents a hydrogen bond (H-bond) interaction. Only those H-bonds that occur between Complex II residues and flutolanil are shown; H-bonds that occur between residues of Complex II were omitted for clarity. Bound cofactors, and a bound fumarate molecule, were also omitted for clarity.

A number of Q-site inhibitors are used as fungicides and interest in their use against nematodes is growing^27^,^28^,^29^. For example, flutolanil has been shown to inhibit complex II from the parasitic nematode *Ascaris suum in vitro*, and a co-crystal structure of flutolanil with this complex has been solved^30^,^31^. We rendered an image of this crystal structure and highlighted the corresponding 14 orthologous residues that are mutated in the wact-11-resistant *C. elegans* mutants (Fig. 4a). Despite residing in three distinct proteins, all the 14 residues cluster around the Q-site where flutolanil is bound. Furthermore, of the 12 residues that are within 4 angstroms of flutolanil's central mass, four are mutated in our wact-11-family resistant mutants (Fig. 4b). Finally, the most frequently mutated residue in our screen is in SDHC-1's R74, which corresponds to *Ascaris'* R89 of SDHC that likely makes electrostatic contacts with the benzene ring of flutolanil's 2-trifluoromethyl-benzamide group^30^,^31^. Like flutolanil, wact-11 also has a 2-trifluoromethyl-benzamide group, and all wact-11 family members have the benzamide moiety. Taken together, our observations suggest that the wact-11-family kills nematodes by binding the Q-site of complex II, and consequently disrupts the interaction of ubiquinone with the complex. Furthermore, the viability of the resistant mutants suggests that the missense mutations alter the Q-site in a way that preserves its function.

We directly tested whether wact-11 and wact-12 can inhibit the enzymatic activity of wild-type *C. elegans* complex II *in vitro* (see Methods). We found that both molecules could inhibit complex II activity with IC50s of 7.4 nM and 5.7 nM, respectively (Table 2, Supplementary Fig. 5). We also tested whether these molecules could inhibit complex II from two independently isolated wact-11-resistant mutants that each harbour the SDHC-1(R74K) missense mutation. We found that complex II from these mutants is insensitive to the molecules up to the highest concentration tested (10 μM; Table 2). As a control, we tested whether wild-type and mutant complex II activity could be inhibited by malonate, which inhibits SDHA-1's succinate-binding activity. We found that malonate inhibits the wild-type complex II to a similar extent as the mutant enzymes (Table 2, Supplementary Fig. 5). These results provide evidence that: (i) mutations in complex II confer resistance to the wact-11-family and (ii) wact-11-family members kill worms by specifically inhibiting complex II at the Q-site.

### A SAR analysis reveals potent wact-11 analogues

We performed a focused structure–activity relationship (SAR) analysis with 16 purchasable analogues of the wact-11 family to better understand the structural elements that impact its bioactivity. We first tested the ability of the analogues to inhibit complex II activity *in vitro*. In general, the presence of an electron-withdrawing group at the 2' position of the benzamide benzene ring favours complex II inhibition because the absence of such a group decreases the inhibition by at least 86-fold relative to wact-11 (see wact-11b and wact-11k in Table 2). This observation is consistent with the proposed binding mechanism of flutolanil (see above). The position of the trifluoromethyl group is also important because relocating it to the 4' position (see wact-11 g in Table 2) decreases complex II inhibition by at least 1,300-fold. The R_2_ phenyl ring of wact-11 promotes complex II inhibition because removing it (see wact-11i) decreases inhibition 44-fold. Finally, having chloride groups attached to the R_2_ phenyl ring promotes complex II inhibition. For example, the two most potent analogues, wact-11 f and wact-11 m, have IC_50_ values of 1 nM, which is over 7-fold lower than that of wact-11, and both of these compounds have electron-withdrawing groups at the 2′ position of their benzamide benzene rings, as well as chloride groups at the 2′ and 4′ positions of their R_2_ phenyl rings.

We next tested the *in vivo* potency of the analogues, and found a positive correlation between the *in vitro* IC_50_ and the *in vivo* LD_100_ (dose lethal to 100% of animals tested) values (Table 2; Pearson's correlation coefficient=0.85), further strengthening the argument that complex II is the *in vivo* target of the wact-11-family. The difference between the *in vitro* and *in vivo* potencies reported in Table 2 is likely due to the resistance of intact *C. elegans* worms to the accumulation of exogenous small molecules^32^. Notably, the two most potent analogues, wact-11 f and wact-11 m, have LD_100_ values in *C. elegans* of 0.469 μM (Table 2). Thus, our SAR analysis revealed nematicides with submicromolar potency.

### Few known complex II inhibitors kill *C. elegans*

In addition to flutolanil, we found other commercial complex II Q-site inhibitors that have structural similarity to the wact-11-family (Supplementary Fig. 6). In particular, fluopyram is structurally similar to wact-11 and has recently been developed as part of a crop spray used to kill parasitic nematodes of plants^22^,^23^. We tested fluopyram and the other structurally related compounds, along with established complex II inhibitors that are structurally unrelated to the wact-11-family (Supplementary Fig. 6), for their ability to kill *C. elegans*. None of these complex II inhibitors are nematicidal up to the highest concentrations tested (120 μM), except for fluopyram and benodanil, which have LD_100_ values of 0.469 μM and 120 μM, respectively (Table 2; Supplementary Fig 7). We found that the wact-11-resistant mutants also resist the lethality induced by fluopyram and benodanil (Supplementary Fig. 7), suggesting that these molecules also target complex II *in vivo*. We also tested the ability of the Q-site inhibitors to inhibit *C. elegans* complex II *in vitro*. Only fluopyram had a potent IC_50_ (1.8 nM), which is almost 2-fold less inhibiting than our two most potent wact-11-family analogues, wact-11 f and wact-11 m.

### The wact-11 family fails to inhibit mammalian complex II

The evidence we have presented supports the idea that mitochondrial complex II is the *in vivo* target of the wact-11-family in nematodes. Because the wact-11 family kills nematodes but not human cells, we hypothesized that mammalian complex II is insensitive to these compounds. We tested this hypothesis by assaying the ability of wact-11, wact-12 and wact-11 f to inhibit murine complex II *in vitro*. Mouse complex II was insensitive to the highest concentrations tested (10 μM) for all three of these compounds (Table 2; Supplementary Fig. 5).

To better understand the phylogenetic selectivity of the wact-11 family, we inspected the conservation among the SDH subunits in nematodes and vertebrates (Supplementary Fig. 8a). First, we found that 12 of the 14 resistance-conferring residues are conserved in all 10 nematode species examined. Of the two that diverge, SDHC-1's C78 diverges in only one of the 10 species and SDHD-1's A97 diverges in only two species. In each case, the substitutions are conservative. These observations suggest that nematodes may in general be sensitive to the wact-11-family of compounds. Second, we analysed the conservation of the 14 residues among seven vertebrate sequences and found that five of the 14 residues are divergent in all vertebrates examined with four of these substitutions being nonconservative (Supplementary Fig. 8b). Given that mutations of any single one of these residues is sufficient to confer resistance to the wact-11-family, it is reasonable to infer that the vertebrate substitutions at these positions will confer resistance to the wact-11 family of nematicides.

## Discussion

Two factors may have discouraged the use of *C. elegans* as a primary high-throughput screening tool to identify novel anthelmintics. First, it is not a parasite and therefore lacks many of the adaptations required for parasitism and the potential anthelmintic targets associated with those processes^16^. Second, the rapid evolution of species within phylum nematoda suggests that *C. elegans* may have essential gene products that may function differently or not exist in some parasitic species^33^. Hence, many of the compounds that kill *C. elegans* may not be effective against parasitic nematodes. However, our work has revealed that molecules that do kill *C. elegans* are more than 15 times more likely to kill parasitic nematodes compared with randomly selected molecules. Given that parasitic nematodes are difficult to screen in high throughput, these results make pre-screening with *C. elegans* an attractive option to increase the throughput of future anthelmintic discovery campaigns.

As expected, not all molecules that kill *C. elegans* are effective against the parasitic models that we tested. In addition, by first screening in *C. elegans*, we have certainly missed molecules that are effective at killing parasitic nematodes but are ineffective in *C. elegans*. However, the speed and ease at which molecules can be screened using *C. elegans* may outweigh the disadvantages it carries as a primary screening system. In principle, *C. elegans* can be used to evaluate hundreds of thousands of molecules at multiple concentrations at a fraction of the cost and time that would be required with most parasitic nematode models.

The extent to which our hits have broad-spectrum activity against distantly-related nematodes is unknown. However, *C. elegans*, *C. oncophora* and *H. contortus* are all clade V nematodes and so molecules that are active against all three of these species are more likely to have broad activity against multiple species in this clade, which include many important parasites of humans and animals^34^.

Using forward small molecule screens to identify anthelmintic leads is a powerful approach because it makes no assumptions about what kind of protein makes a good target. Furthermore, these screens have the potential to yield phylum-specific compounds with unexpected and conserved targets that may not have been considered in target-based searches. Our discovery of the activity and target of the wact-11 family provides a good proof-of-principle for the utility of *C. elegans* as a pre-screening model system.

Multiple lines of evidence show that the wact-11 family targets the conserved complex II of the electron transport chain *in vivo*. First, a good correlation exists between complex II *in vitro* inhibition and *in vivo* potency. Second, out of 33 mutants that resist the wact-11-family, 32 have missense mutations in residues that surround the ubiquinone-binding pocket of complex II. By contrast none of the 36 strains that resist unrelated molecules have mutations in complex II. Third, *in vitro* assays show that the wact-11-family can inhibit complex II from wild-type worms but not from worms that have a mutation in complex II that confers resistance to these molecules *in vivo*. Together, these results indicate that the wact-11 family kills *C. elegans* through its inhibition of complex II.

Our screens have revealed that phylogenetically selective bioactivity is highly dependent upon molecular structure. A systematic analysis of close structural analogues revealed 17 pairs of molecules whose phylogenetic bioactivity profile becomes restricted to nematodes with only small alterations in structure. In addition to the 30 groups of anthelmintic lead structures we describe above, the 275 *C. elegans*-lethals comprised 61 structural groups, 33 of which do not contain a single molecule that kills nematodes selectively. However, given the structure–bioactivity analyses presented above, it may be possible to identify structural analogues that specifically kill nematodes for at least some of these structural groups, raising the total number of potential anthelmintic lead structural classes beyond 30.

Of the 30 distinct groups of anthelmintic leads that we have uncovered, we have attempted to screen for resistance against 16 representative molecules and have failed to generate resistance against all but three. The reason behind this is unclear. Typically, we use ethyl methanesulfonate as a mutagen in our genetic screens, which is biased towards inducing G/C to A/T transitions^35^ and therefore limits the type of non-synonymous mutations that are induced. This is unlikely to explain the lack of success, however, since we also carried out resistance screens using the *N*-ethyl-*N*-nitrosourea mutagen^35^, which produces a variety of nucleotide transitions and transversions, but was equally unsuccessful in yielding resistant mutants (Supplementary Table 1). Instead, we think it more likely that for most lethal molecules, there is no single mutation in the respective target that is capable of conferring resistance. This explanation might be especially true for molecules that inhibit an essential target. It may be that only rare essential targets (like complex II) can be mutated to disrupt the efficacy of an inhibitor without disrupting the target's activity below a viable state. Alternatively, it is possible that many lethal molecules may have multiple essential targets. If true, the generation and isolation of a single mutant animal that has all of the targets mutated to confer resistance would be an exceptionally rare event.

The inability to generate resistant mutants against these molecules has two implications. The first is trivial in that approaches aside from genetics will have to be exploited to identify the target(s) of these molecules. The more important implication is that if the evolution of resistance in the lab can foretell evolution in the field, and there is good evidence for this^3^,^5^,^6^,^7^,^8^,^13^,^14^, then perhaps the converse is true and that the set of anthelmintic leads for which we are unable to generate resistance should be high priority compounds for further development. Regardless, the evolution of parasitic resistance does not immediately negate the usefulness of an anthelmintic. For example, *C. elegans* mutants that resist the effects of benzimidazoles, imidazothiazoles and cyclooctadepsipeptides can be readily generated in the lab^14^,^36^, yet these molecules have been effective in the field for many years despite the eventual emergence of resistance.

## Materials and methods

### Chemical sources

The sources for the chemicals and chemical libraries used in our preliminary screens are indicated in Supplementary Data 1. The wact-11-family and structural analogues were purchased from ChemBridge Corporation. The established complex II inhibitors were purchased from Sigma-Aldrich, with the exception of atpenin A5 and harzianopyridone, which were purchased from Enzo Life Sciences, and thifluzamide, which was purchased from AnGene.

### *C. elegans* strains and culture methods

All the animals were cultured using standard methods at 20 °C (ref. 37), unless otherwise indicated. The N2 (wild-type) strain of *Caenorhabditis elegans* was obtained from the *C. elegans* Genetics Center (University of Minnesota).

### *C. elegans* liquid-based chemical screening

The 96-well liquid-based chemical screening assay was adapted from an established RNAi screening protocol^38^. Briefly, saturated HB101 *Escherichia coli* culture was concentrated 2-fold using NGM (nematode growth media) containing 3 mg ml^−1^ NaCl, 2.5 mg ml^−1^ peptone, 5 μg ml^−1^ cholesterol, 1 mM CaCl_2_, 1 mM MgSO_4_ and 25 mM KH_2_PO_4_. A total of 40 μl of NGM+HB101 was dispensed into each well of a 96-well plate, and chemicals were pinned into the wells using a pinning tool with a 300 nl slot volume (V&P Scientific). Approximately 20 synchronized first larval-stage (L1) worms, in 10 μl of M9 buffer (see ref. 24 for the recipe), were then added to each well. The synchronized L1s were obtained from an embryo preparation (see ref. 24 for the protocol) performed the previous day. The final concentration of dimethyl sulfoxide (DMSO) in the wells was 0.6% v/v. A dissection microscope was used to visualize the wells either 5 or 6 days post worm deposition and any obvious chemical-induced phenotypes were noted.

For our preliminary screens, 50,596 out of 67,012 compounds were assayed in liquid at a 60 μM concentration, and the other 16,416 molecules were screened on solid media at 25 μM (see ref. 24 for a description of our solid-based screening method). From these screens, 672 compounds that induced at least a partially penetrant phenotype were re-ordered and arrayed into a 10-plate ‘worm active' or ‘wactive' library. One hundred and eighty-two molecules chosen at random from the 67,012 compounds were also included in the wactive library, along with many DMSO control wells distributed across the plates.

Using the liquid-based assay, the re-ordered wactive library was re-screened in worms at 7.5, 30 and 60 μM concentrations. Chemicals that induced an obvious phenotype were classified as follows: ‘very strong' molecules induced 100% lethality at 7.5 μM, ‘strong' molecules induced 100% lethality at 30 μM, ‘medium' molecules induced 100% lethality at 60 μM, ‘weak' molecules induced incompletely penetrant lethality at 60 μM and ‘PEP' molecules induced a non-lethal post-embryonic phenotype such as dumpy (Dpy) or uncoordinated (Unc). The 275 *C. elegans*-lethals that are referred to in the text are made up of the ‘very strong', ‘strong' and ‘medium' molecules.

The 96-well liquid-based assay was also used for all follow-up dose-response experiments in this work. These experiments were carried out in quadruplicate, and the total number of worms in each well was counted 6 days after worm deposition.

### *C. oncophora* and *H. contortus* chemical screens

Fresh cattle and sheep faeces containing eggs of an ivermectin-resistant strain of *C. onchophora*^39^ and the MHco3(ISE) strain of *H. contortus*^40^,^41^, respectively, were kindly supplied by Dr Doug Colwell and Dawn Gray (Lethbridge Research Station, Agriculture and Agri-Food Canada). Experimental infections used to generate this material were carried out using established methods^39^,^41^, and were approved by the Lethbridge AAFC Animal Care committee and conducted under animal use license ACC1407. Cattle faeces containing *C. onchophora* eggs were stored anaerobically at 4 °C for a maximum of 3 weeks, whereas sheep faeces containing *H. contortus* eggs were stored at 20 °C for no more than 48 h before harvesting eggs for use. Eggs were isolated from faeces using a standard saturated salt flotation method^42^ immediately before each egg hatch assay. Approximately 100 eggs suspended in 100 μl of water were added to each well of a 96-well plate, and the wactive library chemicals were screened at two different concentrations (7.5 and 60 μM, 0.6% DMSO v/v). Baseline egg hatch rates were determined in DMSO control wells ∼48 h after the initial set-up of the assay by the addition of iodine tincture to stop development. Plates having DMSO control wells with hatch rates >70% were assayed on a semi-quantitative gradient of ‘–' to ‘+++', where ‘–' wells had a hatch rate of <10%, and ‘+++' wells had a hatch rate close to wild type (usually >80%). A dissection microscope was used for visualization purposes. Chemicals were considered bioactive if they consistently had a ‘–' in more than one trial, with ‘very very strong' assigned to compounds that were 90–100% lethal at 7.5 μM in all replicates, ‘very strong' if the same was true at 60 μM, ‘strong' if replicates had a hatch rate between 10 and 50%, ‘medium' if replicates had a 50–80% hatch rate and ‘weak' if only one replicate was between 50 and 80% hatch rate. A molecule was considered ‘lethal' if it exhibited ‘very very strong' or ‘very strong' bioactivity.

### Zebrafish culture and chemical screening

Wild-type (AB) zebrafish embryos were collected in E3 solution (5 mM NaCl, 0.17 mM KCl, 0.33 mM CaCl2, 0.33 mM MgSO4) immediately after fertilization and arrayed at three embryos per well in 96-well plates, 200 μl per well. Wild-type (AB) zebrafish were originally obtained from Zebrafish International Resource Center (University of Oregon). Chemicals were added to each well at 6 h post fertilization at a final concentration of 10 μM (0.5% v/v DMSO), with each plate containing nine wells of DMSO controls. Embryos were examined for mortality and observable developmental phenotypes at 24 and 48 h post fertilization using an Olympus SZX10 Brightfield microscope. Phenotypes examined were death, developmental delay, reduced pigmentation, cranial oedema or cardiac defects (slow/absent heart rate, abnormal heart size). Each compound was screened in duplicate, with only phenotypes appearing in both replicates associated with a given compound. Any compound producing multiple, distinct phenotypes across replicates (for example, cardiac defects in one replicate, mortality in another) was labelled ‘toxic'. A molecule was considered ‘lethal' to zebrafish if it induced ‘death' or ‘toxicity'.

### HEK293 cell culture and chemical screening

HEK293T cells (Attisano Lab, University of Toronto) were maintained in Dulbecco's Modified Eagle's Medium (Gibco) supplemented with 10% FBS (DMEM-10% FBS). Cells at a concentration of 50 cells μl^−1^ were seeded into 96-well plates at a final volume of 100 μl (∼5,000 cells per well). Chemicals from the wactive library were added to the wells for a final concentration of 60 μM (0.6% DMSO v/v). The wactive library plates were screened at least in triplicate. Cell proliferation was determined using a bromodeoxyuridine (BrdU) assay kit (Exalpha Biologicals Inc.). The BrdU was added 2 days after chemical addition. Fixation and denaturation was performed ∼16–18 h later. Anti-BrdU antibody was added, and incorporated BrdU was detected using a horseradish peroxidase-conjugated goat anti-mouse antibody. The BrdU incorporation signal was measured at 450/550 nm for the amount of conversion of tetra-methylbenzidine that is proportional to the amount of BrdU incorporated. For each replicate of each plate, the average signal for the DMSO control wells was calculated. For each well in the plate (including the DMSO control wells), the signal was normalized by dividing by the average DMSO signal. The mean and standard deviation for the population of 960 normalized sample signals of the 10-plate wactive library were calculated and found to be 0.6 and 0.44, respectively. A molecule was considered ‘lethal' if its normalized signal had a magnitude <0.16 (that is, a value less than the s.d. subtracted from the mean).

### Cheminformatics

Chemical structures as supplied by the manufacturers were analysed using the ChemAxon calculator (http://www.chemaxon.com). Specifically, number of hydrogen bond donors, number of hydrogen bond acceptors, mass, atom count, rotatable bond count, logP (a measure of hydrophobicity), Van der Waals surface area, polar surface area, Van der Waals volume and refractivity were computed using default parameters. In the case of salts, all properties were calculated on the largest molecule. Pairwise similarity scores were calculated as the Tanimoto coefficient of shared FP2 fingerprints using OpenBabel (http://openbabel.org). An FP2 fingerprint is a linear fragment of a molecule, containing one to seven atoms. Each pair of compounds to be analysed for similarity were evaluated for presence or absence of any of thousands of possible FP2 fingerprints, and the Tanimoto coefficient represents the number of fingerprints in common between the two compounds divided by the total number of fingerprints present in both compounds. Network visualization for Fig. 2b was performed using Cytoscape^43^.

### Forward genetic screens for resistant mutants

Our forward genetic screens were carried out as previously described^9^,^24^. In brief, wild-type parental (P0) worms were mutagenized in 50 mM ethyl methanesulfonate for 4 h, or in 0.5 mM *N*-ethyl-*N*-nitrosourea for 4 h, as previously described. Tens of thousands of synchronized L1s from either the F1 (progeny) or F2 (grand-progeny) generations were dispensed onto 10 cm MYOB agar plates (see ref. 24 for how to prepare MYOB/agar media) containing an ∼100% penetrant lethal dose of the nematicide. Candidate-resistant mutants are those worms that can grow in the presence of the chemical. Candidates were picked onto solid MYOB plates without any added chemical, and 12 of their progeny were individually re-tested on a 100%-penetrant lethal dose of the nematicide. Those candidates that re-tested were subsequently homozygosed as previously described^9^,^24^.

### Whole-genome sequencing of wact-11-family-resistant mutants

A total 100 μl of packed worms were harvested in a 15 ml conical tube and washed three times with M9 buffer. The worms were then incubated in 6 ml of M9 buffer for 1 h on a nutating shaker at 20 °C. The worms were then washed once with 1 × phosphate-buffered saline (PBS) buffer. The tube was centrifuged and the PBS buffer was aspirated without disturbing the worm pellet. The worms were flash frozen in liquid nitrogen and ground with a pestle until the pellet defrosted. The DNeasy Blood and Tissue Kit (Qiagen) was used to lyse the worm cells and purify the genomic DNA. The Nextera DNA Sample Preparation Kit (Illumina) was used to generate the genomic DNA libraries for sequencing. Individual libraries were quantified with quantitative PCR with reverse transcription using KAPA standards. Multiplexed libraries were sequenced on an Illumina HiSeq2500, paired end reads, 125 bp × 125 bp, using version 4 reagents and flow cells.

Sequence variants were identified using a BWA-GATK pipeline. Briefly, the 125-bp sequencing reads were examined for sequence quality using FastQC (http://www.bioinformatics.babraham.ac.uk/projects/fastqc/) and bases lower than a quality threshold of 30 were trimmed off using Trimmomatic^44^. Reads were aligned to the *C. elegans* N2 reference genome (release W220) using BWA-mem^45^. Alignments were sorted by coordinate order and duplicates removed using Picard (http://picard.sourceforge.net). Before variant calling, reads were processed in Genome Analysis Tool Kit (GATK) v2.5 (ref. 46) for indel realignment and base quality score recalibration, using known *C. elegans* variants from dbSNP build 138 (http://www.ncbi.nlm.nih.gov/SNP/). GATK HaplotypeCaller was used to call variants, and results were filtered for a phred-scaled Q score >30. Finally, called variants were annotated using Annovar^47^ to obtain a list of exonic variants for each sample.

### *Ascaris suum* complex II structure rendering

PyMOL^48^ was used to generate the images in Fig. 4a,b, using the crystal structure 3VRB (downloaded from the Protein Data Bank). H-bonds were identified by performing the ‘find>polar contacts' action in PyMOL^48^, using the default settings.

### Mitochondria isolation from worms

HB101 *E. coli* cells (*C. elegans* Genetics Center, University of Minnesota) from a 1 l saturated culture were pelleted by centrifugation at 2,500*g* for 10 min, and then resuspended in 50 ml complete S-medium (see ref. 37 for recipe). In all, 450,000 synchronized first larval-stage worms, in M9 buffer, were added to the S-media/HB101 suspension and were grown to adulthood over 3.5 days at 20 °C with shaking at 200 r.p.m. Worms were collected in 6 × 15 ml conical tubes and washed eight times with M9 buffer. The worms from the six tubes were combined into one 15 ml conical tube, and resuspended in 15 ml M9 buffer. One millilitre aliquots of the worm suspension were distributed to each of the 15 × 1.5 ml Sarstedt microcentrifuge tubes. The worms were pelleted by centrifugation at 800*g*, and the M9 buffer was aspirated without disrupting the pellet. A total 600 μl of cold isolation buffer A (250 mM sucrose, 10 mM Tris (pH 7.5), 1 mM EDTA, 1 mM PMSF) was added to each tube. A total 300 μl of cold glass beads was added to each tube. The tubes were cooled on ice for 10 min, and the worms were disrupted by bead beating 6 × 30 s, with 1 min cooling intervals. The tubes were spun down at 1,000*g* for 10 min at 4 °C and the supernatants were transferred to a single 15 ml conical tube on ice. The homogenate was centrifuged at 1,000*g* for 10 min at 4 °C. The supernatant was transferred to a new cold tube, and centrifuged at 16,000*g* for 10 min at 4 °C. The pellet was washed twice by re-suspending gently in 5 ml of cold isolation buffer B (250 mM sucrose, 10 mM Tris (pH 7.5), 1 mM EDTA). After the final wash, 310 μl aliquots of the mitochondrial suspension were distributed across 16 microcentrifuge tubes and the tubes were centrifuged at 16,000*g* for 10 min at 4 °C. The supernatant was aspirated and the pellets were snap frozen in liquid nitrogen and stored at −80 °C until needed.

### Mitochondria isolation from mouse liver

The livers from three 8–10-week-old C57Bl/6 female mice (Charles River) were removed, collected in 1.5 ml microcentrifuge tubes, flash frozen in liquid nitrogen and stored at −80 °C until needed. Just before mitochondria isolation, the livers were weighed, placed into a 10-cm petri dish and 10 ml isolation buffer A was added per gram of liver tissue. The tissue was finely minced with a razor blade, transferred to a glass Dounce homogenizer and homogenized by 10 strokes on ice. The homogenate was centrifuged at 1,000*g* for 10 min at 4 °C. The supernatant was collected and centrifuged at 1,000*g* for 10 min at 4 °C. The supernatant was again collected and centrifuged at 16,000*g* for 10 min at 4 °C. The pellet was resuspended in 20 ml of isolation buffer B and centrifuged at 16,000*g* for 10 min at 4 °C. The pellet was resuspended in 20 ml of isolation buffer B, and 1 ml aliquots of the resuspension were distributed into 20 × 1.5 ml microcentrifuge tubes. The aliquoted resuspensions were centrifuged at 16,000*g* for 10 min at 4 °C. The supernatant was aspirated, the pellet was flash frozen in liquid nitrogen and stored frozen at −80 °C until needed. The mice used for experimentation were housed and used in accordance with ‘the use and care of experimental animals' guidelines. The animal protocols were reviewed and approved by the Animal Care Committee of the University of Toronto.

### Cell-free Complex II functional assay

Mitochondrial pellets were thawed on ice and resuspended in 300 μl of cold isolation buffer B. Using the BCA assay^49^, the protein concentration of the mitochondria suspension was determined and was subsequently diluted to a concentration of 0.2 mg ml^−1^ with cold isolation buffer B. Complex II enzymatic assays were carried out in 96-well flat bottom plates. A total 150 μl of complex II assay buffer (1X PBS, 0.35% BSA, 20 mM succinate, 240 μM KCN, 60 μM DCIP, 70 μM decylubiquinone, 25 μM antimycin A, 2 μM rotenone) containing dissolved compounds at the desired concentration, or DMSO (2.4% v/v) alone for control purposes, was added to each well. All of the compounds tested were dissolved in DMSO, except malonate, which was dissolved in water, so DMSO was omitted from the malonate control wells. Five microlitres of the 0.2 mg ml^−1^ mitochondria suspension was added to each well and mixed by pipetting up and down five times. Absorbance for each well was measured at 600 nm using a SpectraMax Plus 384-well Microplate Reader, combined with SoftmaxPro software at 30-s intervals over a 35-min time period. Absorbance versus time was plotted for each well and enzyme activity was calculated as the slope of the line defined by the points ranging from 4 to 10 min. Per cent activity was calculated by dividing the enzyme activity of the chemical-treated wells by that of the DMSO control wells. The per cent activity plotted in Supplementary Fig. 5 is the average of four technical replicates.

## Additional information

**How to cite this article:** Burns, A. R. *et al*. *Caenorhabditis elegans* is a useful model for anthelmintic discovery. *Nat. Commun.* 6:7485 doi: 10.1038/ncomms8485 (2015).

## Supplementary Material

Supplementary Figures and Supplementary Table Supplementary Figures 1-8 and Supplementary Table 1

Supplementary Data 1 Chemical screening summary sheet.

Supplementary Data 2 Raw data for whole genome sequencing.

Supplementary Data 3 Wact-11-family resistant mutants summary sheet.

## Figures and Tables

**Table 1:** Complex II residue changes for the wact-11-family-resistant strains.

**Number of strains**	***C. elegans*** **mutated gene**	***C. elegans*** **residue change**	***Ascaris suum*** **orthologous residue**	**Human orthologous residue**
1	*sdhb-1*	P145L	P127	P131
2	*sdhb-1*	H146Y	H128	H132
6	*sdhb-1*	P211L	P193	P207
1	*sdhb-1*	I260N	I242	I246
3	*sdhc-1*	T66I	T81	P64
1	*sdhc-1*	G71E	G86	I69
8	*sdhc-1*	R74K	R89	R72
1	*sdhc-1*	G77D	G92	G75
1	*sdhc-1*	C78Y	C93	I76
1	*sdhc-1*	G133E	G148	G131
1	*sdhc-1*	F136S	F151	H134
1	*sdhd-1*	H84Q	H95	H98
4	*sdhd-1*	D95N	D106	D109
1	*sdhd-1*	A97T	G108	V111
1	?			
**33 total**				

**Table 2:** Complex II IC_50_ and *in vivo* LD_100_ values for wact-11-family molecules and known complex II inhibitors.

**Compound**	R_1_*	R_2_*	Species	**Strain**	**SDHC residue change**	***In vitro*****Complex II IC**_**50**_ **(nM)**†	***In vivo*****LD**_**100**_ **(μM)**‡
wact-11	2-CF_3_	Ph (4'-Cl)	*C. elegans*	N2	None	7.4	7.5
				RP2674	R74K	>10,000	>120
				RP2698	R74K	>10,000	>120
			*M. musculus*	C57Bl/6	None	>10,000	ND
wact-12	2-I	OPh (4'-CH_2_CH_3_)	*C. elegans*	N2	None	5.7	7.5
				RP2674	R74K	>10,000	>120
				RP2698	R74K	>10,000	>120
			*M. musculus*	C57Bl/6	None	>10,000	ND
wact-11f	2-CF_3_	Ph (2'-Cl, 4'-Cl)	*C. elegans*	N2	None	1.0	0.469
				RP2674	R74K	>10,000	>120
				RP2698	R74K	>10,000	>120
			*M. musculus*	C57Bl/6	None	>10,000	ND
wact-11a	2-CF_3_	Ph	*C. elegans*	N2	None	26.1	60
wact-11i	2-CF_3_	CH_2_CH_3_	*C. elegans*	N2	None	327.2	>120
wact-11g	4-CF_3_	Ph (4'-Cl)	*C. elegans*	N2	None	>10,000	>120
wact-11p	2-I	Ph (4'-Cl)	*C. elegans*	N2	None	5.8	7.5
wact-11e	2-I	Ph (4'-F)	*C. elegans*	N2	None	15.3	60
wact-11d	2-Br	Ph (4'-Cl)	*C. elegans*	N2	None	7.5	1.875
wact-11m	2-Br	Ph (2'-Cl, 4'-Cl)	*C. elegans*	N2	None	1.0	0.469
wact-11j	2-Br	CH_2_CH_3_	*C. elegans*	N2	None	269.6	120
wact-11c	2-F	Ph (4'-Cl)	*C. elegans*	N2	None	170.1	60
wact-11b	-	Ph (4'-Cl)	*C. elegans*	N2	None	632.9	>120
wact-11k	-	CH_2_CH_3_	*C. elegans*	N2	None	>10,000	>120
wact-12b	2-I	OPh (4'-CH_3_)	*C. elegans*	N2	None	7.7	7.5
wact-12c	2-I	SPh (4'-CH_3_)	*C. elegans*	N2	None	132.9	>120
wact-12d	2-I	SPh (4'-Cl)	*C. elegans*	N2	None	128.5	>120
wact-12e	2-Br	OPh (4'-Cl)	*C. elegans*	N2	None	7.8	30
Benodanil			*C. elegans*	N2	None	186.2	120
Boscalid			*C. elegans*	N2	None	549.5	>120
Carboxine			*C. elegans*	N2	None	>10,000	>120
Diazoxide			*C. elegans*	N2	None	>10,000	>120
Fenfuran			*C. elegans*	N2	None	5,279.0	>120
Fluopyram			*C. elegans*	N2	None	1.8	0.469
Flutolanil			*C. elegans*	N2	None	311.2	>120
Harz			*C. elegans*	N2	None	>10,000	>120
Thifluzamide			*C. elegans*	N2	None	3,819	>120
TTFA			*C. elegans*	N2	None	>10,000	>120
Atpenin A5§			*C. elegans*	N2	None	1,678	>120
			*M. musculus*	C57Bl/6	None	593.3	ND
Malonate||			*C. elegans*	N2	None	4.9 × 10^6^	>120
				RP2674	R74K	4.2 × 10^6^	ND
				RP2698	R74K	4.2 × 10^6^	ND

Harz, harzianopyridone; ND, not determined; TTFA, thenoyltrifluoroacetone.

^*^The wact-11-family core structure, with the positions of the R_1_ and R_2_ groups indicated, is shown in Fig. 3

^†^The inhibitory curves used to generate the complex II IC_50_ values can be found in Supplementary Fig. 5.

^‡^The *in vivo* LD_100_ value is defined as the lowest concentration at which no viable animals are visible in four out of four replicate wells, 6 days after 20 first larval-stage worms are deposited. The LD_100_ was determined from a 4-fold dilution series from 120 to 0.00183 μM, and including 60 μM.

^§^For mouse complex II experiments, atpenin A5 was used as a positive control.

^||^For the RP2698 and RP2674 complex II experiments, malonate was used as a positive control.
