# Author Correction: *Caenorhabditiselegans* is a useful model for anthelmintic discovery

**DOI:** 10.1038/s41467-020-17617-3

**Published:** 2020-07-24

**Authors:** Andrew R. Burns, Genna M. Luciani, Gabriel Musso, Rachel Bagg, May Yeo, Yuqian Zhang, Luckshi Rajendran, John Glavin, Robert Hunter, Elizabeth Redman, Susan Stasiuk, Michael Schertzberg, G. Angus McQuibban, Conor R. Caffrey, Sean R. Cutler, Mike Tyers, Guri Giaever, Corey Nislow, Andy G. Fraser, Calum A. MacRae, John Gilleard, Peter J. Roy

**Affiliations:** 1 The Donnelly Centre for Cellular and Biomolecular Research, University of Toronto, Toronto, Ontario, Canada M5S 3E1; 2 Department of Molecular Genetics, University of Toronto, Toronto, Ontario, Canada M5S 1A8; 3 Cardiovascular Division, Brigham and Women’s Hospital, Harvard Medical School, and Harvard Stem Cell Institute, Boston, Massachusetts, 02115 USA; 4 Department of Medicine, Harvard Medical School, Boston, Massachusetts 02115 USA; 5 Department of Comparative Biology and Experimental Medicine, Faculty of Veterinary Medicine, University of Calgary, Calgary, Alberta Canada T2N 4Z6; 6 Department of Biochemistry, University of Toronto, 1 King’s College Circle, Toronto, Ontario Canada M5S 1A8; 7 Center for Discovery and Innovation in Parasitic Diseases and Department of Pathology, University of California, San Francisco, California 94158 USA; 8 Center for Plant Cell Biology, Department of Botany and Plant Sciences, University of California, Riverside, California 92521 USA; 9 Institute for Research in Immunology and Cancer, University of Montreal, Montreal, Quebec Canada H3T 1J4; 10 Department of Pharmaceutical Sciences, University of British Columbia, Vancouver, British Columbia Canada V6T 1Z3; 11 Department of Pharmacology and Toxicology, University of Toronto, Toronto, Ontario Canada M5S 1A8

Correction to: *Nature Communications* 10.1038/ncomms8485, published online 25 June 2015.

The original version of this Article contained an error in the spelling of the author Luckshi Rajendran, which was incorrectly given as Luckshika Rajendran. This has not been corrected in the PDF and HTML versions of the Article.

