# Supplementary material for: Caenorhabditis elegans is a useful model for anthelmintic discovery
Source: Nat Commun. 2015 Jun 25;6:7485. doi: 10.1038/ncomms8485 (PMC4491176; doi:10.1038/ncomms8485)
Supplement: Supplementary Figures and Supplementary Table — Supplementary Figures 1-8 and Supplementary Table 1 [file ncomms8485-s1.pdf]

Supplementary Figure 1.

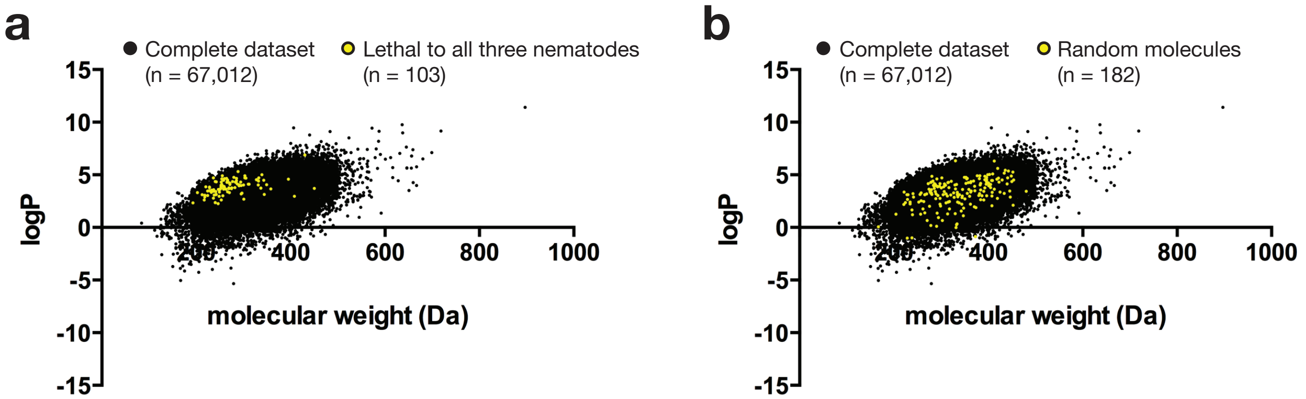

Supplementary Figure 1. Nematicides are generally small and hydrophobic. **(a)** Plot of logP versus molecular weight for the complete set of 67,012 molecules screened in *C. elegans*; the molecules that are lethal to all three nematode species tested are highlighted in yellow. **(b)** Plot of logP versus molecular weight for the complete set of 67,012 molecules screened in *C. elegans*; the set of 182 randomly-selected molecules is highlighted in yellow.

Supplementary Figure 2.

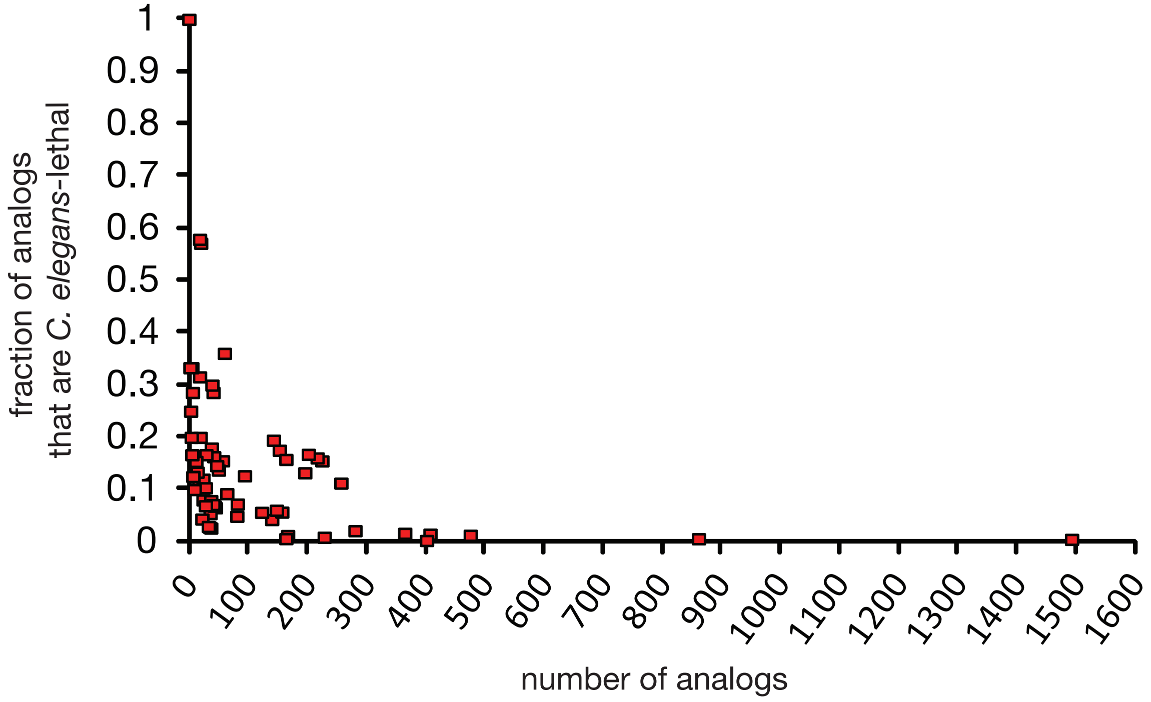

Supplementary Figure 2. Most group 2 wactives are in structural analog families for which more than 10% of the analogs are lethal to *C. elegans*. The 67 individual data points in the plot represent the structural analog families for each of the 67 group 2 wactives. The structural analog family for each group 2 wactive is composed of the individual group 2 compound itself, along with all of the molecules in the 67,012-compound dataset that have a pair-wise Tanimoto/FP2 structural similarity of 0.55 or greater with the group 2 wactive. The fraction of analogs within the structural analog family that is lethal to *C. elegans* is plotted on the y-axis. The number of analogs in the family is plotted on the x-axis.

Supplementary Figure 3.

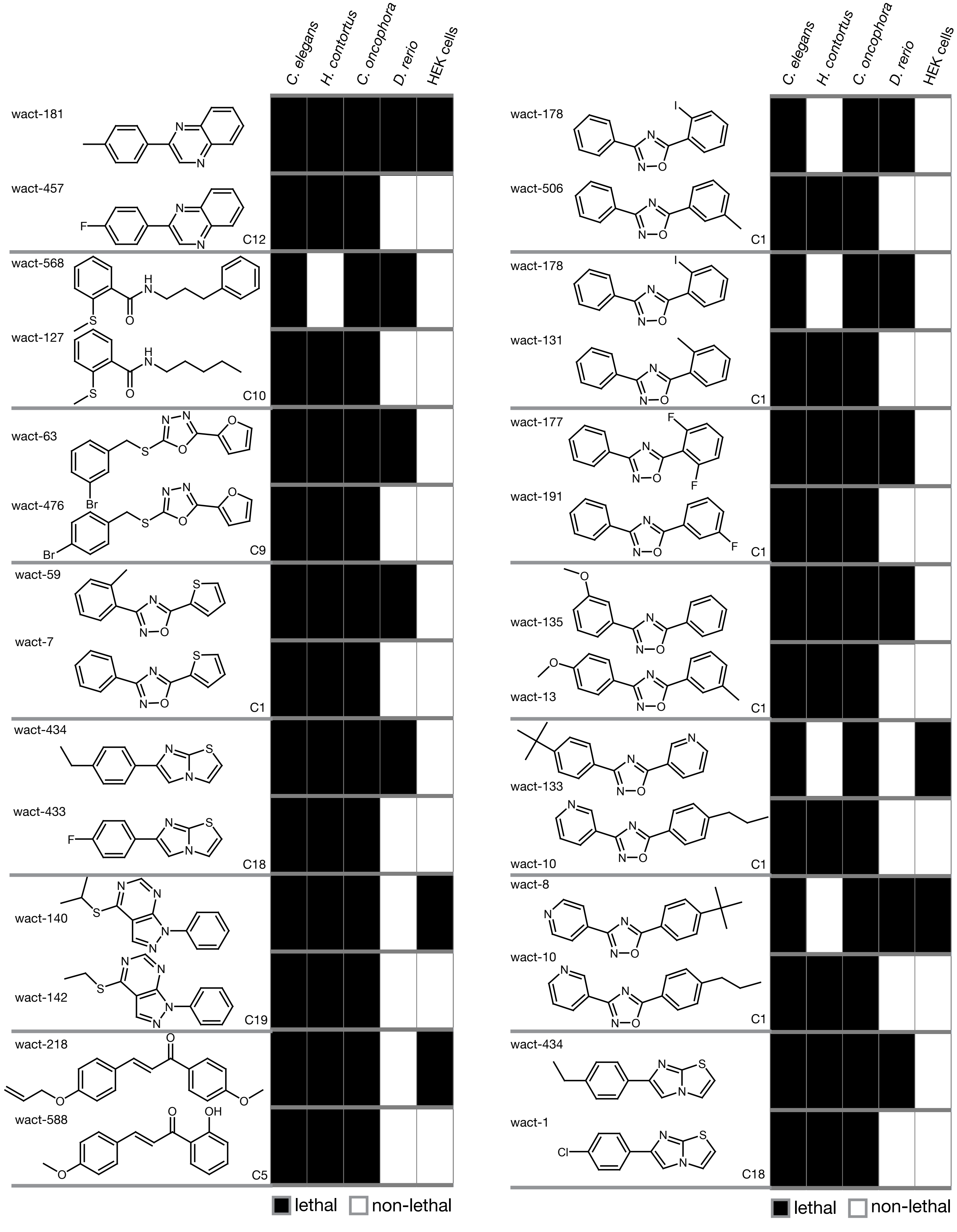

Supplementary Figure 3 (continued).

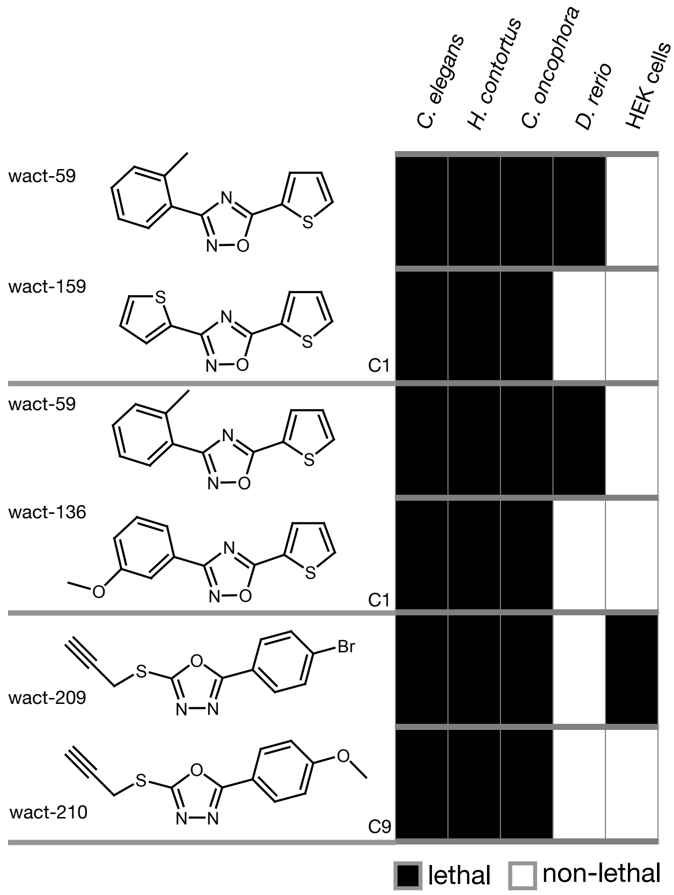

Supplementary Figure 3. Small changes in structure can alter the phylogenetic selectivity of small-molecule-induced lethality. The cross-species lethality profiles for the seventeen pairs of molecules having Tanimoto/FP2 similarity scores greater than 0.8, and for which one molecule of the pair is in group 2 (nematode-restricted lethality) and the other is in group 3 (lethal to a vertebrate system). The network cluster to which each pair belongs is indicated (see Fig. 2b for the corresponding cluster). See Methods for a description of the small molecule screening assays, and the definition of lethality for each organism or cell type.

Supplementary Figure 4.

Strain	Genotype	Residue Change	*A. suum* residue	Human residue
RP2813	*sdhb-1 (tr454)*	P145L	P127	P131
RP2776	*sdhb-1 (tr438)*	H146Y	H128	H132
RP2814	*sdhb-1 (tr455)*	H146Y	H128	H132
RP2696	*sdhb-1 (tr405)*	P211L	P193	P207
RP2697	*sdhb-1 (tr406)*	P211L	P193	P207
RP2703	*sdhb-1 (tr411)*	I260N	I242	I246
RP2639	*sdhc-1 (tr359)*	T66I	T81	P64
RP2687	*sdhc-1 (tr399)*	T66I	T81	P64
RP2667	*sdhc-1 (tr386)*	T66I	T81	P64
RP2637	*sdhc-1 (tr357)*	G71E	G86	I69
RP2674	*sdhc-1 (tr393)*	R74K	R89	R72
RP2698	*sdhc-1 (tr407)*	R74K	R89	R72
RP2699	*sdhc-1 (tr408)*	G77D	G92	G75
RP2748	*sdhc-1 (tr423)*	C78Y	C93	I76
RP2682	*sdhc-1 (tr395)*	G133E	G148	G131
RP2702	*sdhc-1 (tr410)*	F136S	F151	H134
RP2700	*sdhd-1 (tr409)*	H84Q	H95	H98
RP2688	*sdhd-1 (tr400)*	D95N	D106	D109
RP2768	*sdhd-1 (tr436)*	D95N	D106	D109
RP2815	*sdhd-1 (tr456)*	A97T	G108	V111
RP2777	tr439	-	-	-
N2	+	-	-	-
N2	+	-	-	-

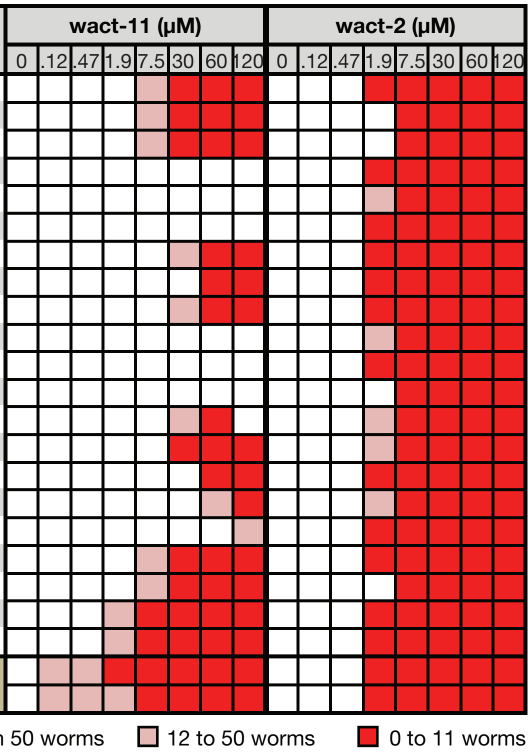

Supplementary Figure 4. Wact-11 resistance profile for 21 wact-11-family resistant mutants. Heat maps of wact-11 dose-response experiments are shown for wild type worms (N2 strain) as well as 21 strains identified as being resistant to either wact-11, wact-12, or wact-127 (see Supplementary Data 3). The dose-response experiments were carried out using a 96-well plate liquid-based assay (see Methods). For RP2777, whole genome sequencing did not reveal an exonic mutation in any of the complex II subunits. Two distinct N2 dose-response experiments were carried out on two different days. Wact-2 dose-response curves were carried out as negative controls.

Supplementary Figure 5.

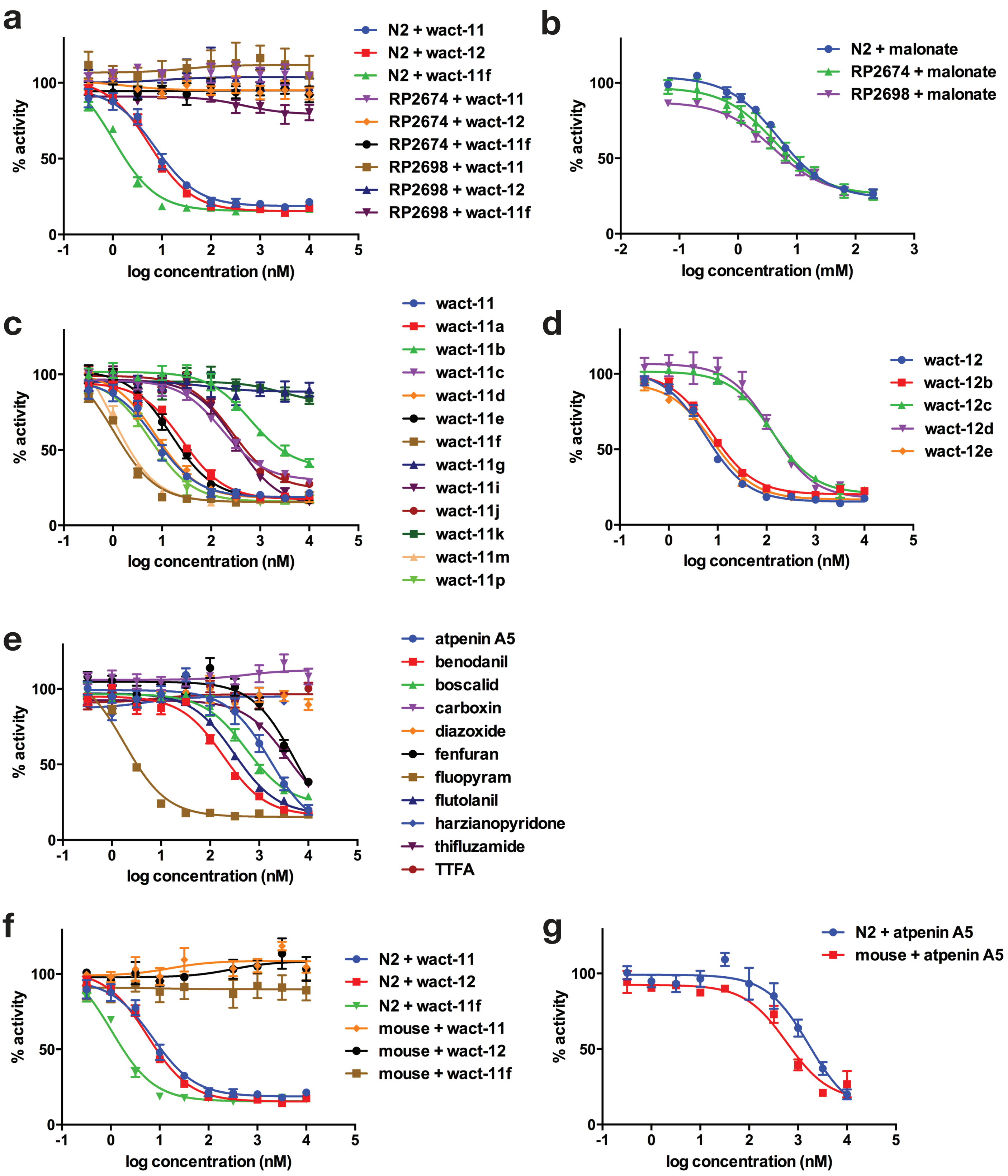

Supplementary Figure 5 (continued).

Supplementary Figure 5 . Complex II inhibitory dose-response curves. Inhibitory dose-response curves for: **(a)** wact-11, wact-12, and wact-11f against complex II from wild type (N2) worms, and the wact-11-family resistant mutants RP2674 and RP2698; **(b)** malonate against complex II from N2 worms, as well as RP2674 and RP2698; **(c)** wact-11 and wact-11 analogs against complex II from N2 worms; **(d)** wact-12 and wact-12 analogs against complex II from N2 worms; **(e)** established complex II inhibitors against complex II from N2 worms; **(f)** wact-11, wact-12, and wact-11f against complex II from N2 worms and mouse liver; **(g)** atpenin A5 against complex II from N2 worms and mouse liver. Error bars represent the standard error of the mean for four replicate trials.

Supplementary Figure 6.

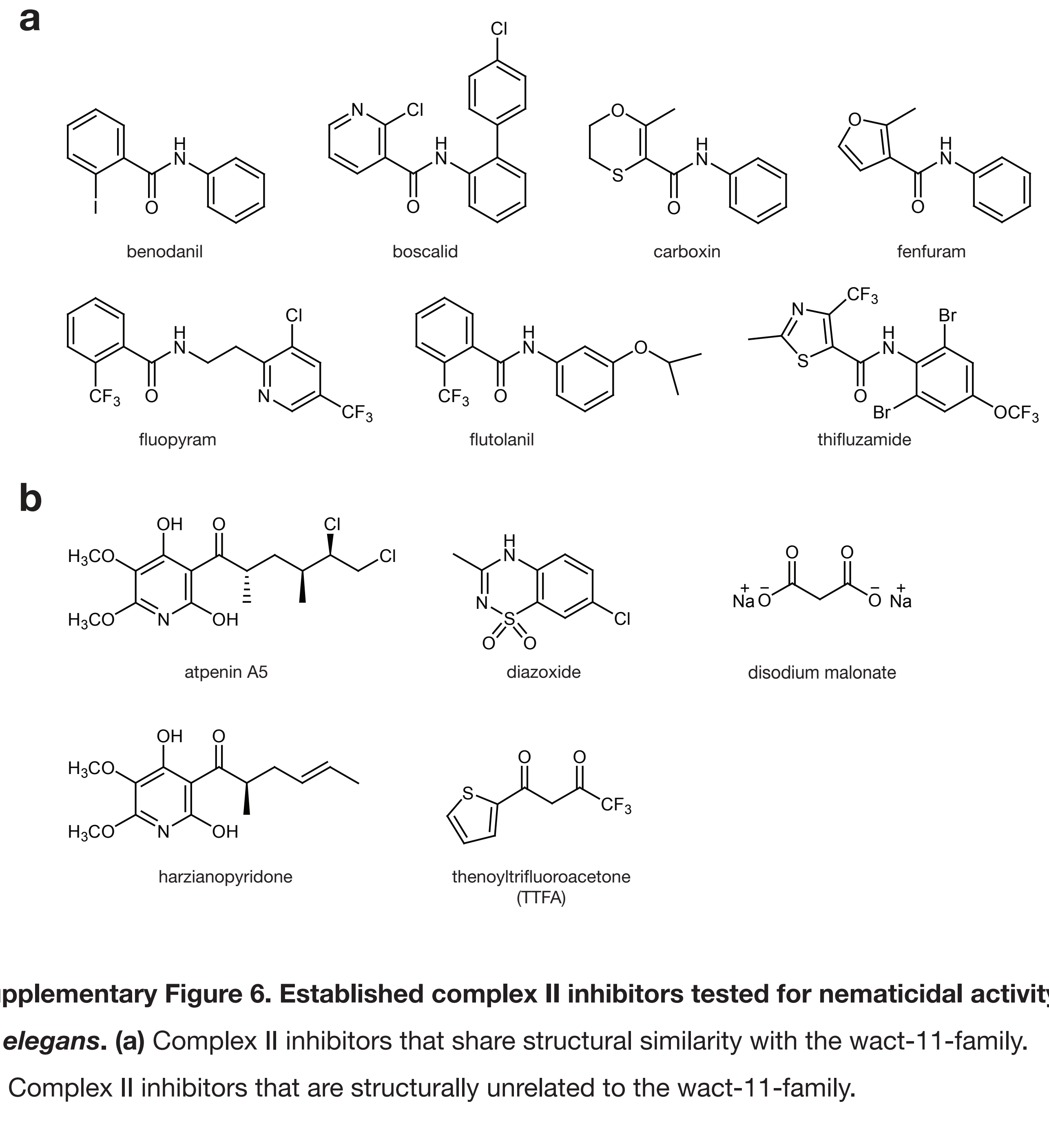

Supplementary Figure 6. Established complex II inhibitors tested for nematicidal activity in *C. elegans*. (a) Complex II inhibitors that share structural similarity with the wact-11-family. **(b)** Complex II inhibitors that are structurally unrelated to the wact-11-family.

Supplementary Figure 7.

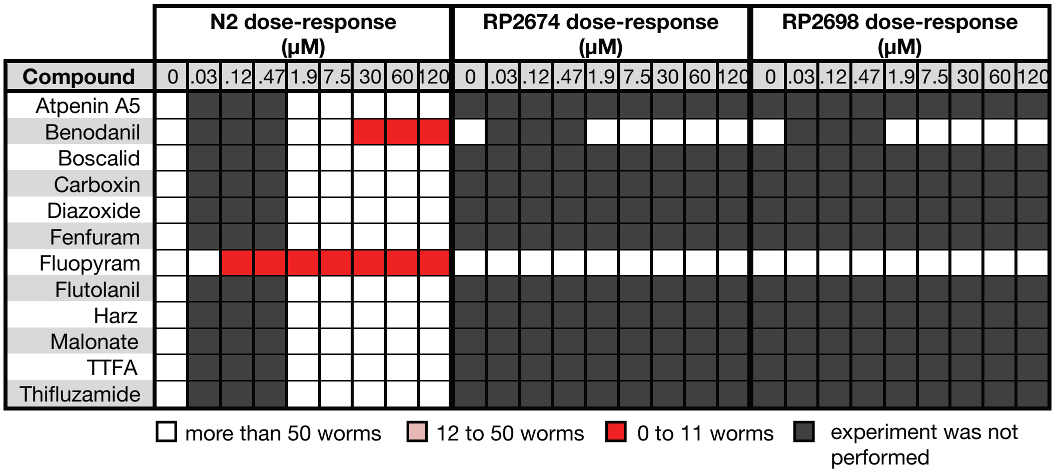

Supplementary Figure 7. Dose-response experiments for known complex II inhibitors. Heat maps are shown for the complex II inhibitor dose-response experiments with wild type worms (N2 strain), as well as two mutant strains, RP2674 and RP2698, isolated as being resistant to wact-12 and wact-11, respectively. The dose-response experiments were carried out using a 96-well plate liquid-based assay (see Methods). The structures of the complex II inhibitors are shown in Supplementary Figure 6. Harz is harzianopyridone and TTFA is thenoyltrifluoroacetone.

Supplementary Figure 8.

a

SDHB

```
                           LY             L            N
   C. elegans [V]    IYPLPHMFV 149  STSCPSYWW 215  KCHTIMNCT 264
  C. briggsae [V]    IYPLPHMFV 133  STSCPSYWW 199  KCHTIMNCT 248
  C. japonica [V]    IYPLPHMFV 133  STSCPSYWW 199  KCHTIMNCT 248
   C. remanei [V]    IYPLPHMFV 134  STSCPSYWW 200  KCHTIMNCT 249
 H. contortus [V]    IHPLPHMYV 127  SSSCPSYWW 193  KCHTIMNCT 242
N. americanus [V]    IYPLPHMFV 65   STSCPSYWW 131  KCHTIMNCT 180
     M. hapla [IV]   IYPLPHMYV 128  STSCPSYWW 194  KCHTILNCT 243
     S. ratti [IV]   IYPLPHMYV 125  STSCPSYWW 191  KCHTILNCT 240
   B. malayi [III]   IYPLPHMFV 128  STSCPSYWW 194  KCHTILNCT 243
     A. suum [III]   IYPLPHMFV 131  SASCPSYWW 197  KCHTIMNCT 246
                                        ** **          * *
```

SDHC

```
                                I    E  K  DY               E  S
   C. elegans [V]    LTVYQPQLTWMLSGFHRISGCVMA 81   HTLNGIRFLGFD 140
  C. briggsae [V]    LTIYQPQLTWMLSGFHRISGCVMA 81   HTLNGIRFLGFD 140
  C. japonica [V]    LTIYQPQLTWMLSGFHRISGCVMA 81   HTLNGIRFLGFD 140
   C. remanei [V]    LTIYQPQLTWMLSGFHRISGCVMA 81   HTLNGIRFLGFD 140
 H. contortus [V]    LTVYKPQLTWMVSGFHRVTGCAMA 77   HTLNGIRFIGFD 136
N. americanus [V]    LTVYQPQLTWMVSGLHRVTGCVMA 79   HTLNGIRFIGFD 138
     M. hapla [IV]   LNIYKPQLTWIISGGHRISGCIMS 80   HSLNGIRFMGFD 139
     S. ratti [IV]   IGIYQMQMTWGLSGLSRISGSIMG 84   HIFNGIRFLGYE 143
   B. malayi [III]   LAVYKPQVTWMVSGFHRMTGCAMA 82   HSLNGIRFIAFD 141
     A. suum [III]   LTIYKPQMTWMVSGLHRVTGCAMA 96   HTLNGIRFIGFD 155
                     *         *  *   *
```

SDHD

```
                         Q          N T
   C. elegans [V]    ALTLHIHWGIHGVVYDYARPYV 101
  C. briggsae [V]    ALTLHIHWGIHGVVYDYARPYV 101
  C. japonica [V]    ALTLHIHWGVHGVVYDYARPYV 101
   C. remanei [V]    ALTLHIHWGIHGVVYDYARPYV 101
 H. contortus [V]    ALTLHIHWGVQGVVQDYARPFV 107
N. americanus [V]    ALTLHIHWGVQGVIQDYARPFV 67
     M. hapla [IV]   AIVMHSHWGLMSVVQDYARPIV 111
     S. ratti [IV]   GCSIHVYLGWHMVTTDYARPFL 108
   B. malayi [III]   AITLHVHWGLHGVLSDYGRAFV 126
     A. suum [III]   ALTLHVHWGVWGVVNDYGRPFV 112
                                    **
```

b

SDHB

```
                     LY             L            N
 C. elegans    IYPLPHMFV 149  STSCPSYWW 215  KCHTIMNCT 264
C. briggsae    IYPLPHMFV 133  STSCPSYWW 199  KCHTIMNCT 248
   S. ratti    IYPLPHMYV 125  STSCPSYWW 191  KCHTILNCT 240
    A. suum    IYPLPHMFV 131  SASCPSYWW 197  KCHTIMNCT 246
   D. rerio    IYPLPHMYV 134  STSCPSYWW 200  RCHTIMNCT 249
M. musculus    IYPLPHMYV 137  STSCPSYWW 203  RCHTIMNCT 252
  G. gallus    IYPLPHMYV 145  STSCPSYWW 211  RCHTIMNCT 260
   O. aries    IYPLPHMYV 135  STSCPSYWW 189  RCHTTTNCT 238
  B. taurus    IYPLPHMYV 135  STSCPSYWW 211  RCHTIMNCT 250
  S. scrofa    IYPLPHMYV 135  STSCPSYWW 211  RCHTIMNCT 250
 H. sapiens    IYPLPHMYV 135  STSCPSYWW 211  RCHTIMNCT 250
                                  ** **          * *
```

SDHC

```
                          I    E  K  DY               E  S
 C. elegans    LTVYQPQLTWMLSGFHRISGCVMA 81   HTLNGIRFLGFD 140
C. briggsae    LTIYQPQLTWMLSGFHRISGCVMA 81   HTLNGIRFLGFD 140
   S. ratti    IGIYQMQMTWGLSGLSRISGSIMG 84   HIFNGIRFLGYE 143
    A. suum    LTIYKPQMTWMVSGLHRVTGCAMA 96   HTLNGIRFIGFD 155
   D. rerio    MTIYKWSVPMAMSISHRGTGIALS 54   HTYNGIRHLAWD 113
M. musculus    LTIYKWSLPMALSVCHRGSGIALS 79   HSLNGIRHLLWD 138
  G. gallus    ISIYKWSLPMAMSITHRGTGVALS 79   HTWNGIRHLVSE 138
   O. aries    ISIYSWSLPMAMSICHRGTGIALS 79   HTWNGIRHLMWD 138
  B. taurus    ISIYGWSLPMAMSICHRGTGIALS 79   HTWNGIRHLMWD 138
  S. scrofa    ITIYRWSLPMAMSICHRGTGIALS 79   HTWNGIRHLIWD 138
 H. sapiens    ITIYSWSLPMAMSICHRGTGIALS 79   HTWNGIRHLMWD 138
               *         *  *   *
```

SDHD

```
                   Q          N T
 C. elegans    ALTLHIHWGIHGVVYDYARPYV 101
C. briggsae    ALTLHIHWGIHGVVYDYARPYV 101
   S. ratti    GCSIHVYLGWHMVTTDYARPFL 108
    A. suum    ALTLHVHWGVWGVVNDYGRPFV 112
   D. rerio    ALTLHGHWGLGQVVTDYVH--- 111
M. musculus    ALTLHSHWGLGQVVTDYVH--- 112
  G. gallus    ALTLHGHWGLGQVITDYVH--- 110
   O. aries    TLTLHSHWGIGQVVTDYVH--- 111
  B. taurus    TLTLHSHWGIGQVVTDYVH--- 111
  S. scrofa    ALTLHGHWGIGQVVTDYVR--- 112
 H. sapiens    ALTLHGHWGLGQVVTDYVH--- 112
                                **
```

Supplementary Figure 8 (continued).

Supplementary Figure 8. Complex II residues that confer wact-11-family resistance when mutated are conserved among nematodes. (a) Sequence alignments for selected regions of Complex II subunits SDHB, SDHC, and SDHD from *C. elegans* and 9 distinct nematode species. The phylogenetic clade for each nematode species is indicated in square parentheses. The *C. elegans* complex II residues that confer wact-11-family resistance when mutated are underlined, and the mutant residues are indicated in red. The residues that are highlighted in grey are identical to the corresponding *C. elegans* residue. The residues that are highlighted in black are distinct from the corresponding *C. elegans* residue. Asterisks denote residues that are within 4 Å of a flutolanil molecule bound at the Q-site of *A. suum* Complex II (see Figure 4). **(b)** Sequence alignments for selected regions of Complex II subunits SDHB, SDHC, and SDHD from *C. elegans*, 3 distinct nematode species, and 7 vertebrate species. Sequence alignments were performed using ClustalW 2.1. All nematode sequences were obtained from WormBase (http://www.wormbase.org). All vertebrate sequences were obtained from the National Center for Biotechnology Information protein database.

Supplementary Table 1. Summary of the genetic screens for wactive-resistant mutants

Chemical ID	Chemical Name	Bioactivity Group #[a]	Generation Screened[b]	Mutagen[c]	Number of Genomes Screened	Number of Resistant Mutants Obtained	Strain Name	Allele Name
6222549	wact-11	2	F1	EMS	1,300,000	13	RP2661	tr380
							RP2668	tr387
							RP2689	tr401
							RP2696	tr405
							RP2697	tr406
							RP2698	tr407
							RP2699	tr408
							RP2700	tr409
							RP2702	tr410
							RP2703	tr411
							RP2704	tr412
							RP2705	tr413
							RP2706	tr414
			F2	EMS	180,000	2	RP2667	tr386
							RP2682	tr395
7003409	wact-12	2	F1	EMS	1,000,000	10	RP2635	tr355
							RP2636	tr356
							RP2669	tr388
							RP2670	tr389
							RP2671	tr390
							RP2673	tr392
							RP2674	tr393
							RP2687	tr399
							RP2688	tr400
							RP2749	tr424
			F2	EMS	240,000	4	RP2637	tr357
							RP2639	tr359
							RP2672	tr391
							RP2683	tr396
7962412	wact-127	2	F1	EMS	980,000	8	RP2748	tr423
							RP2768	tr436
							RP2776	tr438
							RP2777	tr439
							RP2778	tr440
							RP2813	tr454
							RP2814	tr455
							RP2815	tr456
7640928	wact-86	3	F1	EMS	1,600,000	2	RP2809	tr452
							RP2962	tr482

			F2	EMS	100,000	0		
			F1	ENU	1,200,000	1	RP2878	tr457
9013615	wact-154	1	F1	EMS	1,600,000	0		
			F2	EMS	16,000	2	RP2758	tr433
							RP2759	tr434
9036812	wact-190	1	F1	EMS	200,000	0		
			F2	EMS	1,100,000	33	RP2785	tr441
							RP2786	tr442
							RP2787	tr443
							RP2788	tr444
							RP2796	tr445
							RP2797	tr446
							RP2798	tr447
							RP2799	tr448
							RP2800	tr449
							RP2801	tr450
							RP2802	tr451
							RP2896	tr458
							RP2897	tr459
							RP2898	tr460
							RP2899	tr461
							RP2900	tr462
							RP2901	tr463
							RP2902	tr464
							RP2903	tr465
							RP2904	tr466
							RP2905	tr467
							RP2906	tr468
							RP2907	tr469
							RP2908	tr470
							RP2909	tr471
							RP2940	tr472
							RP2941	tr473
							RP2942	tr474
							RP2943	tr475
							RP2944	tr476
							RP2945	tr477
							RP2956	tr480
							RP2957	tr481
5784060	emb-d	2	F1	EMS	270,000	0		
			F2	EMS	100,000	0		
			F1	ENU	320,000	0		

BTB05020	emb-q	1	F1	EMS	200,000	0
5185411	wact-2	1	F1	EMS	150,000	0
			F2	EMS	80,000	0
5347759	wact-3	2	F1	EMS	270,000	0
			F2	EMS	100,000	0
			F1	ENU	320,000	0
5352487	wact-4	2	F1	EMS	620,000	0
			F2	EMS	150,000	0
5352532	wact-5	2	F1	EMS	80,000	0
			F2	EMS	40,000	0
5419367	wact-6	3	F1	EMS	128,000	0
			F2	EMS	86,000	0
5652963	wact-7	2	F1	EMS	200,000	0
			F2	EMS	86,000	0
5652977	wact-8	3	F1	EMS	100,000	0
5905384	wact-10	2	F1	EMS	80,000	0
			F2	EMS	50,000	0
7632721	wact-13	2	F1	EMS	80,000	0
			F2	EMS	100,000	0
7635413	wact-14	2	F1	EMS	310,000	0
			F2	EMS	40,000	0
7721223	wact-16	2	F1	EMS	420,000	0
			F2	EMS	150,000	0
			F1	ENU	320,000	0
5688274	wact-55	2	F1	EMS	190,000	0
			F2	EMS	100,000	0
7662831	wact-89	3	F1	EMS	100,000	0
			F2	EMS	50,000	0
7834045	wact-113	2	F1	EMS	260,000	0
			F2	EMS	100,000	0
7971816	wact-128	2	F1	EMS	100,000	0
			F2	EMS	100,000	0
9000613	wact-139	2	F1	EMS	100,000	0
			F2	EMS	50,000	0
			F1	ENU	320,000	0
9001112	wact-140	3	F1	EMS	100,000	0
			F2	EMS	50,000	0
9010179	wact-145	3	F1	EMS	100,000	0
			F2	EMS	100,000	0
9017143	wact-159	2	F1	EMS	100,000	0
			F2	EMS	50,000	0
9023097	wact-166	3	F1	EMS	100,000	0

9023309	wact-167	2	F1	EMS	80,000	0
			F2	EMS	50,000	0
9024879	wact-169	1	F1	EMS	100,000	0
			F2	EMS	100,000	0
9027529	wact-175	2	F1	EMS	100,000	0
9034543	wact-184	1	F1	EMS	100,000	0
			F2	EMS	100,000	0
9041446	wact-197	2	F1	EMS	100,000	0
			F2	EMS	100,000	0
5784085	wact-220	1	F1	EMS	420,000	0
			F2	EMS	100,000	0
5322542	wact-396	2	F1	EMS	100,000	0
5469460	wact-419	2	F1	EMS	100,000	0
6276968	wact-457	2	F1	EMS	100,000	0
9041091	wact-579	2	F1	EMS	100,000	0
			F2	EMS	50,000	0
			F1	ENU	860,000	0
9039813	wact-614	3	F1	EMS	100,000	0
**TOTALS**			**F1**		**15,478,000**	**34**
			**F2**		**3,718,000**	**41**
			**ALL**		**19,196,000**	**75**

a. Group 1 molecules are lethal to only one or two of the three nematode species tested, but are non-lethal to zebrafish and HEK cells. Group 2 molecules are lethal to all three nematode species, but are non-lethal to zebrafish and human cells. Group 3 molecule are lethal to fish or HEK cells, regardless of their nematode bioactivity profile.
b. F1 refers to the first filial generation, and F2 refers to the second filial generation.
c. EMS = ethyl methanesulfonate, and ENU = *N*-ethyl-*N*-nitrosourea
